# Knowledge Transfer on Complex Social Interventions in Public Health: A Scoping Study

**DOI:** 10.1371/journal.pone.0080233

**Published:** 2013-12-04

**Authors:** Christian Dagenais, Marie Malo, Émilie Robert, Mathieu Ouimet, Diane Berthelette, Valéry Ridde

**Affiliations:** 1 Université de Montréal, Montréal, Québec, Canada; 2 Centre de recherche du Centre hospitalier de l'Université de Montréal (CRCHUM), Montréal, Québec, Canada; 3 Université Laval, Québec, Québec, Canada; 4 Université du Québec à Montréal (UQAM), Montréal, Québec, Canada; 5 Centre de liaison sur l'intervention et la prévention psychosociales (CLIPP), Montréal, Québec, Canada; Katholieke Universiteit Leuven, Belgium

## Abstract

**Objectives:**

Scientific knowledge can help develop interventions that improve public health. The objectives of this review are (1) to describe the status of research on knowledge transfer strategies in the field of complex social interventions in public health and (2) to identify priorities for future research in this field.

**Method:**

A *scoping study* is an exploratory study. After searching databases of bibliographic references and specialized periodicals, we summarized the relevant studies using a predetermined assessment framework. In-depth analysis focused on the following items: types of knowledge transfer strategies, fields of public health, types of publics, types of utilization, and types of research specifications.

**Results:**

From the 1,374 references identified, we selected 26 studies. The strategies targeted mostly administrators of organizations and practitioners. The articles generally dealt with instrumental utilization and most often used qualitative methods. In general, the bias risk for the studies is high.

**Conclusion:**

Researchers need to consider the methodological challenges in this field of research in order to improve assessment of more complex knowledge transfer strategies (when they exist), not just diffusion/dissemination strategies and conceptual and persuasive utilization.

## Introduction

Whether they are endeavoring to improve public health or reduce health-related social inequities, public health officials suggest and initiate increasingly complex interventions [1]. Indeed, the problems they face are most often multidisciplinary because of the diverse social determinants of health [2]. These problems therefore require responses that are adapted to local contexts and involve the participation of a number of people. They differ from clinical interventions, for which the objective is to prevent and treat illness in individuals and which can be handled in a standardized manner [1]. These *complex social interventions*: are based on the presumption that they will produce better results than standard individual interventions; involve the action of several players in the field; consist of a chain process involving several professionals and adapt to the social context in which they occur. The implementation of these interventions is not linear. It uses the bottom-up or top-down model and offers the ability to return to earlier stages of the implementation to adjust and adequately meet the needs of the supporting environment [3]–[5]. To illuminate the complexity of these interventions their characteristics are illustrated in Table 1 [6] by a concrete example based on recent policies in Africa intended to eliminate direct payment for health care. Given the nature of these interventions, knowledge transfer (KT) in such situations is challenging.

**Table 1:** The seven characteristics of complex interventions applied to policies on eliminating healthcare payments in Africa*.

*Complex social interventions …*	*Policies on eliminating direct payment …*
… are theories or consist of several theories.	… seek to reduce the financial burden on households, improve access to health service and provide early recourse to care, and so on.
… involve the participation of numerous stakeholders.	… involve governments, the international community, NGOs, people, health agents, and so on.
… consist of a chain of decision processes.	… assume identification of the problem, formulation of the policy, implementation of the policy by various players, acceptance of the policy by the people and so on.
… are not linear and are subject to feedback loops.	… are transformed and adapted by the action and influence of health agents, patients, decision-makers, and so on.
… are entrenched in several social systems and several contexts.	… are implemented in a number of countries, which have different populations, living with distinct social realities, distinct representations of the world and different healthcare systems.
… are permeable to the influence of other interventions.	… coexist with other healthcare policies or social interventions that influence them.
… are open learning systems.	… are systems in which the health agents, patients, decision-makers and others adapt their practices, behaviors, attitudes, and so on.

* Taken from Ridde et al., 2012.

The popularity of KT has been growing since the 1880s, especially in the health sector. For example, the Canadian Institute of Health Research now funds projects that promote the use of research-based knowledge by potential users. There have also been many systematic and critical reviews of the literature. They generally deal with transfer of knowledge from clinical research, such as the efficiency of strategies that promote knowledge use [7]–[8], including practice guidelines [9]–[10]. None of these critical reviews deal with KT from research involving complex social interventions in public health, despite the major challenge in encouraging interventions based on convincing evidence.

The rapid development of the research field on the use of scientific knowledge has manifested itself in the emergence in recent decades of numerous terms to refer to the concept of “knowledge to action”. In a study of 33 funding organizations in 9 countries, Graham and his colleagues [11] identified 29 different terms used including knowledge transfer, knowledge translation and knowledge mobilization, among others. Most of these terms have been “promoted” by research funding agencies. For example, the Canadian Institutes for Health Research are using the term “knowledge translation”, while the Social Sciences and Humanities Research Council used instead “knowledge mobilization”. Although the definition of these terms may sometimes vary, different words are mostly used to designate more or less the same thing. To avoid this confusion, some organisations are now using the term K* (K star: for knowledge whatever…). In this study, we use the term knowledge transfer (KT), which is still the most frequently used term.

The knowledge transfer strategies are ultimately aimed at the use of knowledge. This study considers three types of knowledge use: instrumental utilization (i.e., changes in behavior or practice), conceptual utilization (i.e., changes in understanding or attitude), and persuasive utilization (i.e., arguments used to influence policies or practices) [12].

With a view to guiding KT research in the field of complex social interventions in public health, we examined existing literature on this topic to obtain an overview of the knowledge available. More precisely, we looked at two distinct aspects: (a) KT strategies and the way they are assessed and (b) the manner in which knowledge utilization is measured. The objectives of this scoping study are (1) to describe the status of research on knowledge transfer strategies in complex social interventions in the public health field and (2) to identify priorities for future research.

## Methods

Our critical review of scientific literature was conducted as a scoping study [13]. This type of review differs from systematic reviews in that the intention is to obtain an overall picture of an issue or field of research in order to assess the feasibility of a systematic review and guide future research: it does not assess the effectiveness of an intervention. In this study, the choice to include or exclude a study was based not on the research specifications, but rather on relevance to the topic in question [13]. We also included gray literature. Our study was divided into four steps: 1) study identification; 2) choice and application of selection criteria; 3) data classification and 4) data analysis.

The review was carried out in accordance with a protocol developed in advance (http://equiperenard.ca/fr/protocole.html). The PRISMA (Preferred Reporting Items for Systematic Reviews and Meta-Analyses) checklist for this paper is presented as Table S1 (supporting information S1).

### Study Identification

First, the following public health databases were consulted: MEDLINE, ERIC, PsycINFO, CINAHL and the French public health database, *Banque de données santé publique*. The search in each of these databases was limited to the period from 1960 to October 2010. Key words included the term “knowledge transfer” and its alternative expression as listed in Graham et al. [11], in English and French. The search strategy adopted for the various databases is described in Table S2 (Supporting information S2). The Tables of Contents of two periodicals, *Evidence & Policy* (2005 to January 2011) and *Implementation Science* (2006 to January 2011) were also thoroughly explored. Next, a snowball strategy was applied to the bibliographies of articles identified in the databases and selected for analysis (the inclusion criteria are provided below). This combination of research strategies was used to find the maximum number of documents on the topic.

### Selection Criteria

The studies selected 1) focused on KT in public health; 2) dealt with the use of research-based knowledge; 3) covered complex social interventions in public health; 4) provided empirical data; 5) were written in English or French; and 6) were published in or after 1960, the decade in which the concept of KT appeared in scientific documentation [14]. Moreover, since the objective was to obtain a complete picture of this field of research, the type of study specification was not considered as a criterion for inclusion or exclusion.

With these criteria in mind, a first sort was done by consulting titles, then document abstracts. Two reviewers worked independently on this sort on the references obtained from the databases. The reviewers agreed 95.7% of the time. Differences were resolved by consensus. A second sort was then done in which the texts selected during the first sort were read in their entirety.

### Data Classification

The articles selected were distributed to three members of the research team who classified the data and then checked all their work. For the specific requirements of this study, an assessment framework was developed [15]. For this framework, the team members looked at the following information: authors, publication year and location, sample type, types of knowledge users, fields of practice, research goals, methods, measuring instruments and important results. The Mixed Method Appraisal Tool (MMAT) developed by Pluye and his collaborators [16] (Table S3: Supporting information S3) was used to evaluate the quality of the articles selected on the basis of the method used. This tool links five types of research specifications (e.g. qualitative) with quality-criteria questions (e.g., does the process for analyzing data allow answering the research question?). All of the conclusions from the use of this framework were checked by at least one member of the team and differences were resolved by consensus. Because it is the only assessment framework that combines evaluation of five types of research specifications, quantitative, qualitative or mixed, this instrument appeared to us to be the most suitable for the study context.

### Data Analysis

From the grouped data, an overall description of all the material was compiled. We compared the different studies and found gaps in research on KT strategies and knowledge utilization in complex social interventions in public health.

## Results

**Figure 1:**
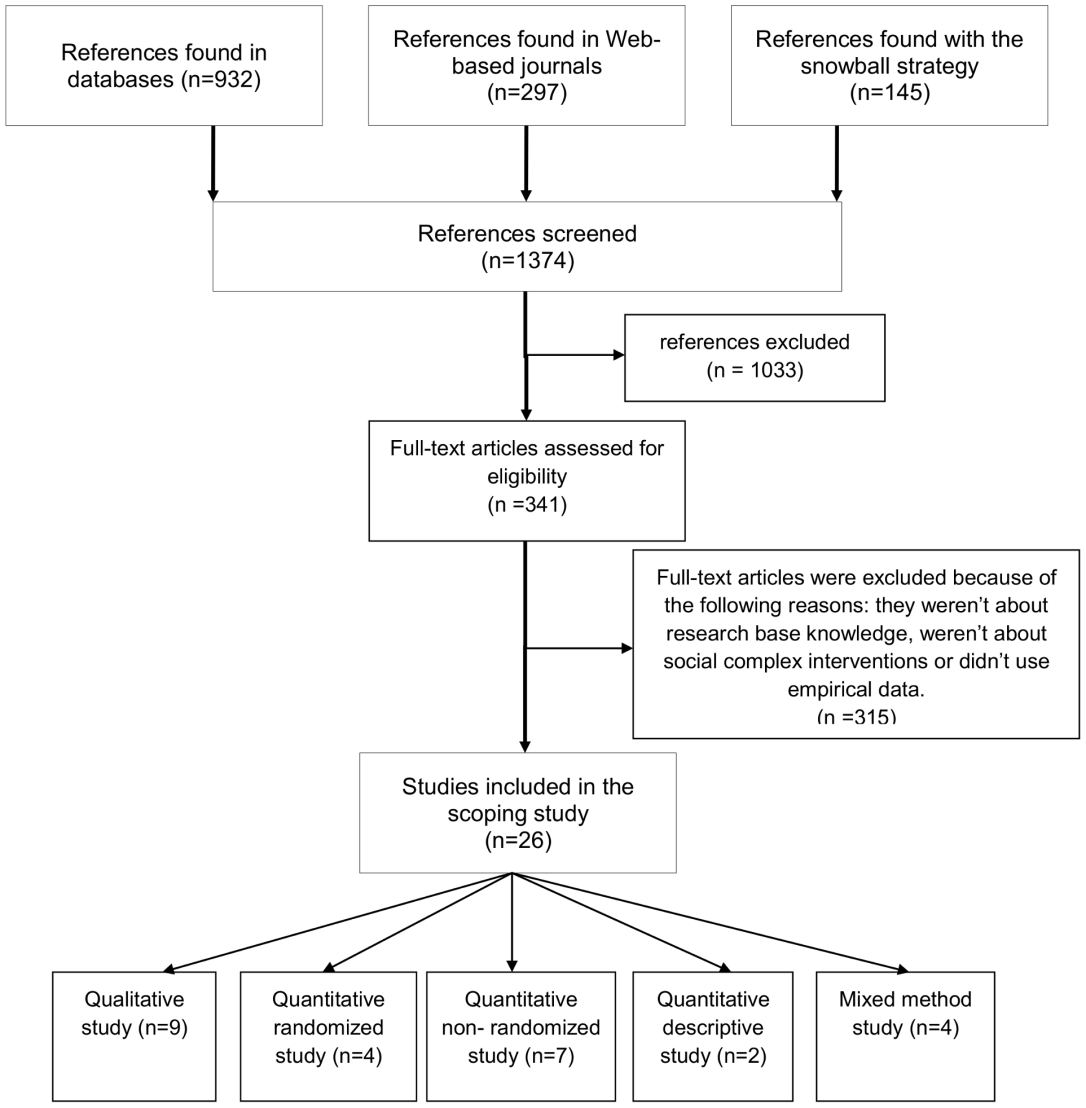
Study identification strategy.

Our research identified 1,374 potentially relevant references (see the PRISMA flowchart Figure 1). Based on the titles and abstracts, 341 articles were read for a more in-depth examination. Of these, 26 empirical studies were included.

### KT Strategies, Recipients and Users

Table 2 provides the characteristics of the studies on KT strategies. The field and type of study, strategies and type of utilization were examined [17], [32]. For the target publics, we differentiated the recipients—individuals or groups directly targeted by the KT strategy—and the publics concerned by the knowledge in question. Table 3 gives the characteristics of the studies that measure knowledge utilization [33], [42]. We examined the field of public health, the type of study specifications, the type of utilization measured, and the knowledge users.

**Table 2:** Characteristics of KT strategy assessment studies included in the scoping study.

Authors	Field	KT Strategy	Types of Use	Recipients	Publics Targeted by the Intervention
Adily, et al. (2009) [16]	Public health (general)	Research partnership	Instrumental utilization	Policy makers Administrators/managers Practitioners Researchers	Not specified
Armstrong, et al. (2007) [17]	Health promotion	Dissemination strategy	Instrumental and persuasive utilization	Practitioners Organizations	Not specified
Brownson, et al.(2007) [18]	Health promotion	Dissemination strategy	Instrumental and conceptual utilization	Administrators/managers Practitioners	Population (United States)
Dobbins, et al.(2009) [19]	Health promotion	Dissemination strategies and knowledge broker	Instrumental utilization	Organizations	Preschoolers/school children/adolescents (Canada)
Kelly, et al.(2000) [20]	Public health (general)	Dissemination strategies	Instrumental utilization	Organizations	Other (United States)
Klein, et al.(2001) [21]	Public health (general)	Training and dissemination program	Instrumental utilization	Administrators/managers Practitioners Organizations	Preschoolers/school children/adolescents (United States)
Kothari, et al.(2005) [22]	Public health (general)	Research partnership	Conceptual utilization	Administrators/managers	Women (Canada)
Lewis, et al.(2005) [23]	Public health (general)	Dissemination strategy	Instrumental utilization	Administrators/managers	Other (United States)
Lia-Hoagberg, et al. (1999) [24]	Public health (general)	Dissemination strategy	Instrumental utilization	Administrators/managers Practitioners	Other (United States)
Manske(2001) [25]	Public health (general)	Liaison centre	Instrumental and conceptual utilization	Administrators/managers Practitioners	Population (Canada)
Masuda, et al.(2009) [26]	Health promotion	Dissemination strategies	Instrumental utilization	Organizations	Population (Canada)
McCormick & Tompkins(1998) [27]	Public health	Dissemination strategies	Instrumental utilization	Organizations	Preschoolers/school children/adolescents (United States)
McFarlane, et al.(2001) [28]	Health services administration/organization of care	Dissemination strategies	Instrumental utilization	Organizations	Patients (United States)
McVey, et al.(2009) [29]	Health promotion	Online training program	Instrumental and conceptual utilization	Practitioners	Preschoolers/school children/adolescents (Canada)
Naylor, et al.(2006) [30]	Health promotion	Project partnership	Instrumental utilization	Administrators/managers Practitioners Researchers Organizations	Preschoolers/school children/adolescents (Canada)
Schinke, et al.(2002) [31]	Public health (general)	Dissemination strategy	Instrumental utilization	Administrators/managers Practitioners	Population (United States)

**Table 3:** Characteristics of research utilization studies included in the scoping study.

Authors	Field	Type of Utilization	Users
Aarons, et al.(2009) [32]	Public health (general)	Instrumental utilization	Practitioners
Dobbins, et al.(2004) [33]	Health policies	Instrumental utilization	Policy makers Administrators/managers
Ir, et al.(2010) [34]	Health administration/organization of care	Instrumental utilization	Policy makers
Lavis, et al.(2002) [35]	Health policies	Instrumental utilization	Policy makers
Mackenzie, et al.(2006) [36]	Health policies	Conceptual utilization	Policy makers
Oh & Rich(1996) [37]	Health administration/organization of care	Instrumental utilization	Policy makers Administrators/managers
Ouimet, et al.(2009) [38]	Health policies	Conceptual and persuasive utilization	Policy makers Administrators/managers
Patton, et al.(1975) [39]	Public health (general)	Instrumental and conceptual utilization	Policy makers
Toomey, et al.(2009) [40]	Public health (general)	Instrumental utilization	Policy makers Administrators/managers
Waddell, et al.(2005) [41]	Health policies	Persuasive utilization	Policy makers

In the articles assessing KT strategies, (Table 2), most of the strategies focus only on diffusion/dissemination: distribution of documentation (adapted or not), e-mailing, or development of Web sites, for example. These activities are sometimes accompanied by telephone assistance or training workshops. The strategies often involve several dissemination activities: distribution of policy briefs and workshops, for example. Only two articles deal with research made in partnership withwith stakeholders. One study discusses the knowledge transfer activities made by a liaison centre and one evaluates a KT strategy involving a knowledge broker. This strategy is designed to support evidence-based decision-making in the organization, management and execution of health services. The studies generally contain few details about the strategies, so relatively little is known about the knowledge concerned, the underlying conceptual or theoretical bases, or their objectives.

As for the knowledge users targetedtargeted, the two categories most often concerned are administrators or managers of organizations and practitioners. Few strategies specifically address policy makers or researchers. On the other hand, many have an organizational component and are intended to bring about a more systemic change. As for the target publics, they are usually the general public in the United States or Canada. Some of the strategies assessed deal with knowledge relating to preschoolers or school children, but few target women or actual patients.

In the articles that measure research utilization, (Table 3), we identified three main categories of users: administrators or managers (i.e., people in an organization), practitioners, and policy makers. Most of the studies deal with research utilization among policy makers; some with utilization by administrators or managers. One discusses research utilization by practitioners. Table 4 lists the number of studies by user type and target public.

**Table 4:** Research users and target public of KT strategies.

Users		Recipients		Target Public	
Policy makers	9	Policy makers	1	General public	4
Administrators/managers	4	Administrators/managers	9	Preschoolers/school children/adolescents	5
Practitioners	1	Practitioners	9	Women	1
		Researchers	2	Patients	1
		Organizations	8	Other/Not specified	5

### What type of use for which field of public health?

We classified the studies according to the public health field and the type of utilization (Table 5). In general, most of the articles dealt with instrumental utilization, particularly in public health and health promotion. It is interesting to note that some studies specifically in these two areas discuss conceptual utilization. Persuasive utilization is very rarely considered in the studies.

**Table 5:** Types of utilization by public health field*.

	Instrumental Utilization	Conceptual Utilization	Persuasive Utilization
Public health (general)	++++++++++**	+++	
Environmental health			
Health policies	++	++	++
Health promotion	+++++++	++	+
Health administration/organization of care	+++		
TOTAL	22	7	3

* Some studies deal with more than one field.

** The number of crosses matches the number of articles.

### Research specifications and study quality

Tables 6 and 7 show the results produced by the Mixed Method Appraisal Tool developed by Pluye et al. [16]. Table 6 shows the relationship between the research specifications and the three types of research utilization. Regardless of the research specifications, the studies deal mainly with instrumental utilization of knowledge, except for the qualitative studies that also take conceptual utilization into account.

**Table 6:** Types of research utilization by type of research specification.

Type of specification	Instrumental Utilization*	Conceptual Utilization	Persuasive Utilization
Qualitative	++++++**	+++	++
Quantitative randomized	++++	+	
Quantitative non-randomized	++++++	++	+
Quantitative descriptive	++		
Mixed	++++	+	

* Categories not exclusive.

** The number of crosses matches the number of articles.

**Table 7:** Research specifications and article quality according to MMAT [15].

Study Type	Criteria	Number of Articles		
		Present	Absent	Not Mentioned
Qualitative (n = 9)	1.1	8	1	0
	1.2	6	2	1
	1.3	5	2	2
	1.4	3	5	1
Quantitative randomized (n = 4)	2.1	2	2	0
	2.2	2	2	0
	2.3	3	1	0
	2.4	2	0	2
Quantitative non- randomized (n = 7)	3.1	6	0	1
	3.2	3	4	0
	3.3	7	0	0
	3.4	6	1	0
Quantitative descriptive (n = 2)	4.1	2	0	0
	4.2	2	0	0
	4.3	0	2	0
	4.4	2	0	0
Mixed method(n = 4)	5.1	4	0	0
	5.2	2	0	2
	5.3	1	3	0

Table 7 shows the research specifications regarding study quality. In decreasing order of frequency, the studies are based on qualitative (9/27), quantitative non-randomized (7/27), quantitative randomized (4/27), mixed methods (4/27) and descriptive (2/27) specifications.

Overall, the quantitative studies are based on data sources and analysis techniques that answer the research question and take the context into account. However, in half of the qualitative studies, the researchers did not specify their *a priori* considerations or the impacts of these assumptions on interpretation of the results.

Most of the quantitative non-randomized studies include participant recruitment mechanisms that reduce selection bias, have a response rate of at least 60% and explain the comparison between the groups sampled. Over half of the studies use measuring instruments whose psychometric properties are not well-documented.

As for the quantitative randomized studies, half do not provide a clear description of the selection method or attrition information, while three-quarters of them have less than 20% missing data.

In the studies using mixed methods, application of the Pluye et al. [16] criteria shows that the specifications chosen are relevant for meeting the research objectives. In addition, half of the studies explain the integration of the qualitative and quantitative results and only one article does not provide the limits of this integration.

Two studies use a quantitative descriptive specification. The sampling strategies appear relevant for answering the research questions, the samples seem representative of the population and the response rates are greater than 60%.

The measuring instruments are rarely described well enough to judge their quality. More specifically, reliability and validity are rarely reported.

## Discussion and Conclusion

### Limits of the study

One limitation is related to the study topic: the definition of *complex social interventions* was particularly difficult to pin down from the literature. On the one hand, this term incorporates two concepts that are still not well-developed, complexity and the social nature of an intervention, which makes it difficult to obtain a precise definition. Articles often comment superficially on the knowledge behind the KT strategy that is being assessed and we sometimes needed to do additional research to determine what this knowledge was. This was not always possible, so we decided to include false positives, i.e., studies for which the knowledge behind the KT strategy does not necessarily involve a complex social intervention. On the other hand, a more restrictive or broader definition of this expression might have made the composition of the pool of articles somewhat different.

With regard to Mixed Method Appraisal Tool analysis of study quality, it would be necessary to contact the authors of the articles when the response to one of the screening questions was incomplete after reading the articles. Owing to lack of resources, we were unable to do this. Our description of quality is therefore based exclusively on the information available in the article and the way in which the authors presented their study.

### Status of research on KT and areas for future research

We found relatively few studies dealing with our research topic, KT strategies for complex social interventions in public health. The definition we chose may have reduced the pool of existing studies. These concepts are recent and the definition is still fuzzy. Furthermore, as we have seen, the vast majority of identified studies have been published over the past ten years; Thus, this field of research is recent, not yet well-developed and involves major methodological challenges for researchers. Since not much knowledge is available on the effectiveness of this type of intervention, it is not surprising that research on knowledge transfer to user groups remains limited.

Neither was it surprising that no studies were found on KT strategies for complex social interventions in environmental health or health administration. It is also possible that the search strategy was unable to track down specialized literature in these areas. KT in public health is therefore a field of study that remains to be explored.

In addition to these contextual comments, we observed that most of the included studies deal with instrumental utilization of knowledge, whether relating to assessment of KT strategies or measurement of knowledge use. Despite the methodological challenges involved in this type of study, we can suppose that they are not as great as for studies of conceptual or persuasive utilization. What method(s) should be preferred? What measurements are appropriate? What measuring instrument(s) should be used? What construct(s) should be studied? As Weiss noted in 1977, future research should pay attention to these questions as a number of authors [11], [43], mention the significance of these types of utilization in complex decision making.

Moreover, the effects of a KT strategy on instrumental utilization can be measured in the short term and is evident in observable behavior, even though knowledge use is a process that can continue in the medium and long terms. Researchers may therefore tend to prefer to study this type of use. When other types of utilization are considered, such as conceptual utilization, the effects are not evident in observable behavior and this makes their study more complex. Since the effects of KT strategies on knowledge use can appear in the short, medium and long terms, the choice of the appropriate time to assess a KT strategy also poses a challenge.

The question of what methods to use to assess KT strategies or measure knowledge use is closely tied to these challenges. Few studies use mixed-method design or descriptive quantitative design. Although descriptive quantitative design may impose limitations on evaluation of this type of relatively complex intervention, the use of mixed-method design appears to us to be worth exploring. Given the complexity of KT strategies and the extent of the potential effects, and considering the added value of these methods in enhancing understanding of a phenomenon, [4], [5], it seems obvious to us that mixed-method studies should be used more.

The issue of study quality is also pivotal. Aside from the aforementioned limitations of using the Mixed Method Appraisal Tool, our results clearly show that it is premature to conduct a systematic review, Cochrane-style for example, on this specific study topic. Firstly, few studies could be included in such a systematic review because of their methodological attributes. Secondly, the methodological weakness of most of the studies would limit the scope of the systematic review results. As for measurement of knowledge use, we noted that the studies devote little explanation to the measuring instruments used, which makes it difficult to evaluate the quality of these instruments and, by extension, the quality of the results produced.

Finally, this study has shown the preponderance of evaluations of strategies limited to diffusion/dissemination. Yet thanks to studies of factors that promote knowledge use, we now know that diffusion and dissemination alone have a very limited impact. It is therefore surprising to see the limited number of more complex strategies implemented [43]–[46]. We now know that one-way, push-pull-type strategies are often insufficient to promote knowledge application [10], [47] compared to two-way strategies [48]. There seems to be a gap between existing knowledge about effective strategies and the strategies that are actually implemented. This being said, the task here was to identify studies that evaluate KT strategies, and not to identify the strategies themselves. We can imagine that more elaborate strategies are now being implemented but have not yet been researched. We also wonder whether the methodological challenges and lack of funding are responsible for limiting researchers' interest in this type of intervention.

Finally, with regard to the second objective of this study, we think that future research on KT strategies involving complex social interventions in public health should consider that::

- Some reflection is required regarding the methodological issues raised by KT studies in general and more specifically on measurement of conceptual and persuasive utilization of knowledge.
- We encourage the organizations involved in KT to describe the conceptual and theoretical bases of their KT strategies. This would help researchers understand the logic behind the KT strategies and assess their plausibility, relevance and validity, which is rarely done.
- Future research should focus on two aspects: assessment of KT strategies used in complex social interventions with explicit descriptions in the articles of what knowledge is involved; and assessment of more complex, elaborate KT strategies (when they exist), not just diffusion/dissemination strategies.
- Lastly, a brief look at the Web shows a lot of activity surrounding knowledge transfer and a multitude of strategies of all kinds that are being used in the world. However, although much has been written on the theory of the potential effects of these strategies and the factors that promote their use, convincing evidence remains limited [43] [49]–[52]. Consequently, we think researchers should endeavor to develop assessment projects for various KT strategies and publish their results so that this knowledge can be utilized.

## Supporting Information

Table S1 **PRISMA checklist.**
(DOCX)

Table S2 **Search Strategy.**
(DOCX)

Table S3 **Mixed Method Appraisal Tool.**
(DOCX)
