# Supplementary material for: Knowledge Transfer on Complex Social Interventions in Public Health: A Scoping Study
Source: PLoS One. 2013 Dec 4;8(12):e80233. doi: 10.1371/journal.pone.0080233 (PMC3851180; doi:10.1371/journal.pone.0080233)
Supplement: Table S2 — Search Strategy. (DOCX) [file pone.0080233.s002.docx]

**Table S2: Search Strategy**

**ERIC, MEDLINE, PsychINFO**

1. (best practice* adj adopt*).mp. [mp=ab, ti, hw, id, ot, nm, an, ui, tc]

2. (evidence adj implement*).mp. [mp=ab, ti, hw, id, ot, nm, an, ui, tc]

3. (evidence adj uptake).mp. [mp=ab, ti, hw, id, ot, nm, an, ui, tc]

4. (evidence adj practice).mp. [mp=ab, ti, hw, id, ot, nm, an, ui, tc]

5. (guideline* adj implement*).mp. [mp=ab, ti, hw, id, ot, nm, an, ui, tc]

6. (information* adj disseminat*).mp. [mp=ab, ti, hw, id, ot, nm, an, ui, tc]

7. (information* adj utili*at*).mp. [mp=ab, ti, hw, id, ot, nm, an, ui, tc]

8. (innovation* adj adopt*).mp. [mp=ab, ti, hw, id, ot, nm, an, ui, tc]

9. (innovation* adj diffus*).mp. [mp=ab, ti, hw, id, ot, nm, an, ui, tc]

10. know-do.mp. [mp=ab, ti, hw, id, ot, nm, an, ui, tc]

11. (knowledge adj action).mp. [mp=ab, ti, hw, id, ot, nm, an, ui, tc]

12. (knowledge adj practice).mp. [mp=ab, ti, hw, id, ot, nm, an, ui, tc]

13. (knowledge adj broker*).mp. [mp=ab, ti, hw, id, ot, nm, an, ui, tc]

14. (knowledge adj communicat*).mp. [mp=ab, ti, hw, id, ot, nm, an, ui, tc]

15. (knowledge adj2 application).mp. [mp=ab, ti, hw, id, ot, nm, an, ui, tc]

16. (knowledge adj diffus*).mp. [mp=ab, ti, hw, id, ot, nm, an, ui, tc]

17. (knowledge adj disseminat*).mp. [mp=ab, ti, hw, id, ot, nm, an, ui, tc]

18. (knowledge adj exchang*).mp. [mp=ab, ti, hw, id, ot, nm, an, ui, tc]

19. (knowledge adj manag*).mp. [mp=ab, ti, hw, id, ot, nm, an, ui, tc]

20. (knowledge adj mobili*).mp. [mp=ab, ti, hw, id, ot, nm, an, ui, tc]

21. (knowledge adj synthesi*).mp. [mp=ab, ti, hw, id, ot, nm, an, ui, tc]

22. (knowledge adj transfer*).mp. [mp=ab, ti, hw, id, ot, nm, an, ui, tc]

23. (knowledge adj transform*).mp. [mp=ab, ti, hw, id, ot, nm, an, ui, tc]

24. (knowledge adj translat*).mp. [mp=ab, ti, hw, id, ot, nm, an, ui, tc]

25. (knowledge adj uptake).mp. [mp=ab, ti, hw, id, ot, nm, an, ui, tc]

26. (knowledge adj utili*ation).mp. [mp=ab, ti, hw, id, ot, nm, an, ui, tc]

27. (linkage adj exchange).mp. [mp=ab, ti, hw, id, ot, nm, an, ui, tc]

28. (research adj "use").mp. [mp=ab, ti, hw, id, ot, nm, an, ui, tc]

29. (research adj action).mp. [mp=ab, ti, hw, id, ot, nm, an, ui, tc]

30. (research adj practice).mp. [mp=ab, ti, hw, id, ot, nm, an, ui, tc]

31. (research adj application).mp. [mp=ab, ti, hw, id, ot, nm, an, ui, tc]

32. (research adj disseminat*).mp. [mp=ab, ti, hw, id, ot, nm, an, ui, tc]

33. (research adj implement*).mp. [mp=ab, ti, hw, id, ot, nm, an, ui, tc]

34. (research adj intervention*).mp. [mp=ab, ti, hw, id, ot, nm, an, ui, tc]

35. (research adj mediat*).mp. [mp=ab, ti, hw, id, ot, nm, an, ui, tc]

36. (research adj transfer*).mp. [mp=ab, ti, hw, id, ot, nm, an, ui, tc]

37. (research adj translat*).mp. [mp=ab, ti, hw, id, ot, nm, an, ui, tc]

38. (research adj utili*ation).mp. [mp=ab, ti, hw, id, ot, nm, an, ui, tc]

39. (science adj communicat*).mp. [mp=ab, ti, hw, id, ot, nm, an, ui, tc]

40. (technolog* adj transfer*).mp. [mp=ab, ti, hw, id, ot, nm, an, ui, tc]

41. 1 or 2 or 3 or 4 or 5 or 6 or 7 or 8 or 9 or 10 or 11 or 12 or 13 or 14 or 15 or 16 or 17 or 18 or 19 or 20 or 21 or 22 or 23 or 24 or 25 or 26 or 27 or 28 or 29 or 30 or 31 or 32 or 33 or 34 or 35 or 36 or 37 or 38 or 39 or 40

42. (change adj behavio?r).mp. [mp=ab, ti, hw, id, ot, nm, an, ui, tc]

43. cooperation.mp. [mp=ab, ti, hw, id, ot, nm, an, ui, tc]

44. effectiveness research.mp. [mp=ab, ti, hw, id, ot, nm, an, ui, tc]

45. evaluation research.mp. [mp=ab, ti, hw, id, ot, nm, an, ui, tc]

46. (feedback adj2 audit).mp. [mp=ab, ti, hw, id, ot, nm, an, ui, tc]

47. gap analysis.mp. [mp=ab, ti, hw, id, ot, nm, an, ui, tc]

48. impact.mp. [mp=ab, ti, hw, id, ot, nm, an, ui, tc]

49. implementation research.mp. [mp=ab, ti, hw, id, ot, nm, an, ui, tc]

50. implementation science.mp. [mp=ab, ti, hw, id, ot, nm, an, ui, tc]

51. measur*.mp. [mp=ab, ti, hw, id, ot, nm, an, ui, tc]

52. outcome*.mp. [mp=ab, ti, hw, id, ot, nm, an, ui, tc]

53. payback.mp. [mp=ab, ti, hw, id, ot, nm, an, ui, tc]

54. process*.mp. [mp=ab, ti, hw, id, ot, nm, an, ui, tc]

55. program*.mp. [mp=ab, ti, hw, id, ot, nm, an, ui, tc]

56. quality assurance.mp. [mp=ab, ti, hw, id, ot, nm, an, ui, tc]

57. quality improvement.mp. [mp=ab, ti, hw, id, ot, nm, an, ui, tc]

58. 42 or 43 or 44 or 45 or 46 or 47 or 48 or 49 or 50 or 51 or 52 or 53 or 54 or 56 or 57

59. community health.mp. [mp=ab, ti, hw, id, ot, nm, an, ui, tc]

60. (complex adj2 intervention*).mp. [mp=ab, ti, hw, id, ot, nm, an, ui, tc]

61. health intervention.mp. [mp=ab, ti, hw, id, ot, nm, an, ui, tc]

62. health policy.mp. [mp=ab, ti, hw, id, ot, nm, an, ui, tc]

63. health promotion.mp. [mp=ab, ti, hw, id, ot, nm, an, ui, tc]

64. population health.mp. [mp=ab, ti, hw, id, ot, nm, an, ui, tc]

65. preventive medicine.mp. [mp=ab, ti, hw, id, ot, nm, an, ui, tc]

66. public health.mp. [mp=ab, ti, hw, id, ot, nm, an, ui, tc]

67. social intervention*.mp. [mp=ab, ti, hw, id, ot, nm, an, ui, tc]

68. 59 or 60 or 61 or 62 or 63 or 64 or 65 or 66 or 67

69. 41 and 58 and 68

1. (research adj utili*ation).mp. [mp=ab, ti, hw, id, ot, nm, ui, tc]

2. (research adj implement*).mp. [mp=ab, ti, hw, id, ot, nm, ui, tc]

3. (research adj "use").mp. [mp=ab, ti, hw, id, ot, nm, ui, tc]

4. (research adj application).mp. [mp=ab, ti, hw, id, ot, nm, ui, tc]

5. (research adj translat*).mp. [mp=ab, ti, hw, id, ot, nm, ui, tc]

6. (research adj transfer).mp. [mp=ab, ti, hw, id, ot, nm, ui, tc]

7. (research adj disseminat*).mp. [mp=ab, ti, hw, id, ot, nm, ui, tc]

8. (research adj intervention*).mp. [mp=ab, ti, hw, id, ot, nm, ui, tc]

9. (research adj action).mp. [mp=ab, ti, hw, id, ot, nm, ui, tc]

10. (knowledge adj translat*).mp. [mp=ab, ti, hw, id, ot, nm, ui, tc]

11. (knowledge adj utili*ation).mp. [mp=ab, ti, hw, id, ot, nm, ui, tc]

12. (knowledge adj transfer).mp. [mp=ab, ti, hw, id, ot, nm, ui, tc]

13. (knowledge adj disseminat*).mp. [mp=ab, ti, hw, id, ot, nm, ui, tc]

14. (knowledge adj mobili*).mp. [mp=ab, ti, hw, id, ot, nm, ui, tc]

15. (knowledge adj diffusion).mp. [mp=ab, ti, hw, id, ot, nm, ui, tc]

16. (knowledge adj action*).mp. [mp=ab, ti, hw, id, ot, nm, ui, tc]

17. (knowledge adj broker*).mp. [mp=ab, ti, hw, id, ot, nm, ui, tc]

18. "knowledge management".mp. [mp=ab, ti, hw, id, ot, nm, ui, tc]

19. "knowledge uptake".mp. [mp=ab, ti, hw, id, ot, nm, ui, tc]

20. "knowledge exchange".mp. [mp=ab, ti, hw, id, ot, nm, ui, tc]

21. (innovation adj adoption).mp. [mp=ab, ti, hw, id, ot, nm, ui, tc]

22. (implementation adj evidence).mp. [mp=ab, ti, hw, id, ot, nm, ui, tc]

23. (evidence adj uptake).mp. [mp=ab, ti, hw, id, ot, nm, ui, tc]

24. 1 or 2 or 3 or 4 or 5 or 6 or 7 or 8 or 9 or 10 or 11 or 12 or 13 or 14 or 15 or 16 or 17 or 18 or 19 or 20 or 21 or 22 or 23

25. (outcome* or measur* or evaluat* or process* or program*).mp. [mp=ab, ti, hw, id, ot, nm, ui, tc]

26. ("public health" or "community health" or "population health" or "health promotion" or "preventive medicine" or "health polic*" or "health intervention*").mp. [mp=ab, ti, hw, id, ot, nm, ui, tc]

27. 24 and 25 and 26

28. limit 27 to yr="1960 -Current"

29. remove duplicates from 28

**Implementation Science**

1. public AND health OR health AND policy OR health AND intervention OR community AND health OR 'health promotion' OR 'population health' OR 'preventive medicine' OR 'social intervention' NOT ‘clinical’ (in title and abstract)
