# Supplementary material for: Knowledge Transfer on Complex Social Interventions in Public Health: A Scoping Study
Source: PLoS One. 2013 Dec 4;8(12):e80233. doi: 10.1371/journal.pone.0080233 (PMC3851180; doi:10.1371/journal.pone.0080233)
Supplement: Table S3 — Mixed Method Appraisal Tool. (DOCX) [file pone.0080233.s003.docx]

**Table S3 : Mixed Method Appraisal Tool** **(Pluye et al., 2011)**

| Types of mixed methods study components or primary studies | Methodological quality criteria (see tutorial for definitions and examples) | Responses | | | |
| --- | --- | --- | --- | --- | --- |
|  |  | Yes | No | Can’t tell | Comments |
| Screening questions  (for all types) | Is there a clear qualitative and/or quantitative research question (or research objective)?  Do the collected data allow answering (meeting) the research question (objective)? E.g., consider whether the follow-up period was long enough for the outcome to occur (concerning longitudinal studies or study components).  Further quality appraisal may be not feasible when the answer is ‘No’ or ‘Can’t tell’ to one or both questions. |  |  |  |  |
| 1. Qualitative | 1.1. Do the data sources, e.g., participants, allow answering (meeting) the research question (objective)? |  |  |  |  |
|  | 1.2. Does the process for analyzing qualitative data allow answering (meeting) the research question (objective)? |  |  |  |  |
|  | 1.3. Is appropriate consideration given to how findings relate to the context, e.g., the setting, in which the data were collected? |  |  |  |  |
|  | 1.4. Is appropriate consideration given to how findings relate to researchers’ influence, e.g., through their interactions with participants? |  |  |  |  |
| 2. Quantitative randomized controlled (trials) | 2.1. Is there a clear description of the randomization (or an appropriate sequence generation)? |  |  |  |  |
|  | 2.2. Is there a clear description of the allocation concealment (or blinding when applicable)? |  |  |  |  |
|  | 2.3. Is there complete outcome data (80% or above)? |  |  |  |  |
|  | 2.4. Is there low withdrawal/drop-out (below 20%)? |  |  |  |  |
| 3. Quantitative non-randomized | 3.1. Are participants recruited in a way that minimized selection bias? |  |  |  |  |
|  | 3.2. Are measurements appropriate (clear origin, or validity known, or standard instrument; and absence of contamination between groups when appropriate) regarding the exposure/intervention and outcomes? |  |  |  |  |
|  | 3.3. In the groups being compared (exposed vs. non-exposed; with intervention vs. without; cases vs. controls), are the participants comparable, or do researchers take into account (control for) the difference between these groups? |  |  |  |  |
|  | 3.4. Is there complete outcome data (80% or above), and, when applicable, an acceptable response rate (60% or above), or an acceptable follow-up rate for cohort studies (depending on the duration of follow-up)? |  |  |  |  |
| 4. Quantitative descriptive | 4.1. Does the sampling strategy allow answering the research question? |  |  |  |  |
|  | 4.2. Is the sample representative of the population under study? |  |  |  |  |
|  | 4.3. Are measurements appropriate (clear origin, or validity known, or standard instrument)? |  |  |  |  |
|  | 4.4. Is there an acceptable response rate (60% or above)? |  |  |  |  |
| 5. Mixed methods | 5.1. Does the mixed methods research design allow answering (meeting) the research question (objective)? |  |  |  |  |
|  | 5.2. Does the integration of qualitative and quantitative data (or results) allow answering (meeting) the research question (objective)?* |  |  |  |  |
|  | 5.3. Is appropriate consideration given to the limitations associated with this integration, e.g., the divergence of qualitative and quantitative data (or results) in a triangulation design? |  |  |  |  |
|  | Criteria for the qualitative component (1.1 to 1.4), and appropriate criteria for the quantitative component (2.1 to 2.4, or 3.1 to 3.4, or 4.1 to 4.4), must be also applied. | | | | |
